# A metastasized hepatocellular carcinoma in the capsule of an undescended testis in the right inguinal area: report of a rare case

**DOI:** 10.1186/s12957-018-1319-4

**Published:** 2018-01-19

**Authors:** Qianhui Li, Xiuying Shi, Chuifeng Fan

**Affiliations:** Department of Pathology, First Affiliated Hospital and College of Basic Medical Sciences of China Medical University, Shenyang, 110001 China

**Keywords:** Cryptorchidism, Hepatocellular carcinoma, Undescended testis, Inguinal region, Case report

## Abstract

**Background:**

Hepatocellular Carcinoma (HCC) is the most common primary carcinoma of the liver, which mainly metastasizes through the portal vein system.

**Case presentation:**

Here, we report an extremely rare case in which HCC metastasized to the capsule of an undescended testis in the right inguinal area of the patient. A tumor approximately 8.8 × 7.0 cm in size was found in the patient’s liver during a health check-up. Initially, it was considered a metastatic tumor because the patient was found to have cryptorchidism, which had been left untreated before he presented to our hospital. The patient underwent a radical orchiectomy via inguinal approach, and the resected testis in the right inguinal region was examined via microscopy. The cancer cells were arranged in nests and showed abundant red or clear cytoplasm and marked nuclear atypia. Immunohistochemical staining showed that the tumor cells were positive for CK, CK8/18, AFP, hepatocyte, GCP3, but negative for PLAP, CD10, CD30, CD34, and vimentin.

**Conclusion:**

According to these findings, the tumor in the inguinal region was considered a metastatic HCC arising from the liver, rather than a seminoma that had originated in the undescended testis. We suggest that during the diagnosis of malignancies, metastatic tumors should always be considered in the differential diagnosis even if the original presentation is at rare metastatic sites or concurrent with other disease(s).

## Background

Hepatocellular carcinoma (HCC) is the most common primary carcinoma of the liver. The most common route by which hepatocellular cell carcinoma metastasizes is through the portal vein system. Distant hematogenous metastases are most frequently seen in the lungs [1], whereas rare distant metastatic sites include the jaw, skull, skin, and mandible [2–5]. Here, we report a rare case of HCC that had metastasized to the capsule of an undescended testis in the right inguinal region of a patient with cryptorchidism. The finding of HCC with histologically and immunohistochemically confirmed metastasis to a cryptorchid testis is itself a rarity. In the literature, there has been an isolated case of HCC with metastasis to the testis [6]; however, to the best of our knowledge, there has been no similar finding involving an undescended testis. Seminomas are more frequently found in the undescended testis [7], and can metastasize to the liver. As the patient had been found to have cryptorchidism before the mass in the liver was observed during a health check-up, the mass was initially considered to be a metastatic tumor from the undescended testis by the examining clinician. However, visualization via microscopy of the undescended testis showed a tumor present in the capsule, which is in accordance with the histological features of HCC. Thus, it is appropriate to consider the mass as a metastasized HCC from the liver, though it is an extremely rare presentation of the disease.

## Case presentation

A 59-year-old man presented to our facility owing to an incidental finding of a liver mass during a routine health check-up a month earlier. He also had a cryptorchid right testis since birth. However, he was asymptomatic. Blood testing determined that α-fetoprotein (AFP) levels were markedly elevated (> 1210 ng/mL). The carcinoembryonic antigen (CEA) level was also slightly elevated (6.01 ng/mL). The levels of carbohydrate antigen 19–9 (CA19–9) and carbohydrate antigen 125 (CA125) were deemed to be within normal ranges. β-HCG was normal (2.3 mIU/ml).

**Fig. 1:**
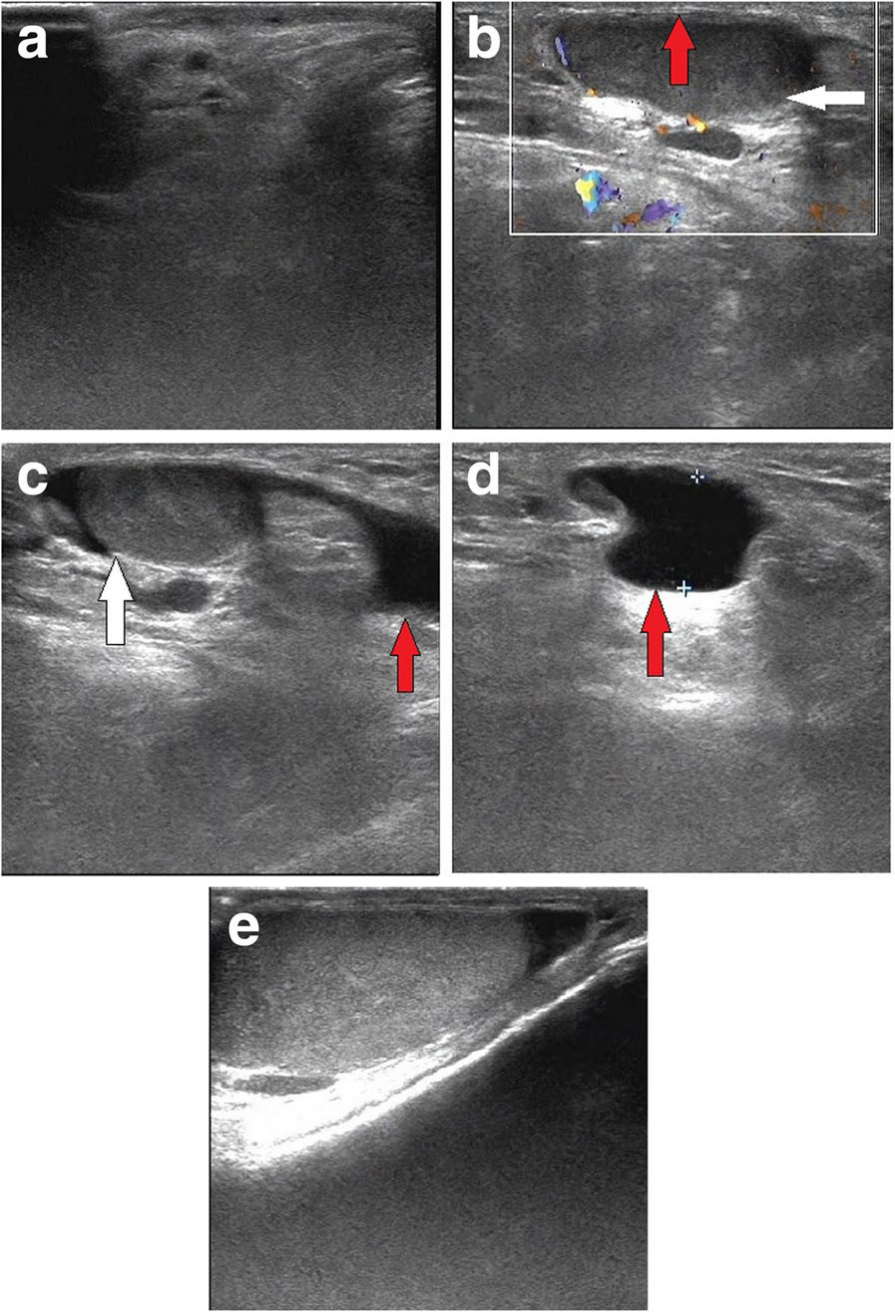
The ultrasonography of the testes. There was no testis in the right scrotum (**a**). There was an undescended testis in the right inguinal area, which was approximately 3.01 cm × 1.09 cm in size (**b**, white arrow). There was a thin layer of tissues with abnormal signal with dense echo in the peripheral part of the testis (red arrow). There was effusion (**c**, **d**, red arrow) adjacent to the testis (**c**, the white arrow). The left testis was seen in the left scrotum and no mass was found (**e**). The left testis was about 3.0 cm and relative small

**Fig. 2:**
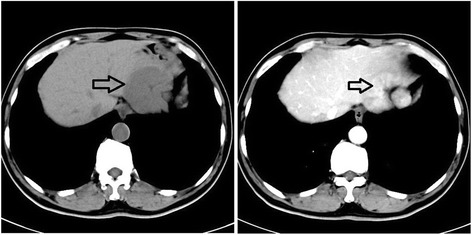
Computed tomography findings of the tumor in the liver. The tumor in the liver was located in the right lobe and was approximately 8.8 × 7.0 cm in size, as indicated by the arrows. The boundary is not clear and the density is uneven

Ultrasound findings of the testes are shown in Fig. 1. Figure 1a shows that there were no testes in the scrotum. Figure 1b shows the undescended testis in the inguinal area, which was approximately 3.01 × 1.09 cm (white arrow) in size. The red arrow highlights that there was a thin layer of tissues with abnormal signal with dense echo in the peripheral part of the testis, which was pathologically confirmed to be cancerous tissues. Figure 1c demonstrates the testis in the inguinal region (white arrow) and the surrounding effusion (red arrow). Figure 1d shows the area of effusion (red arrow). Figure 1e shows the left testis in the scrotum, which was relative smaller and about 3.0 cm. No mass was detected in the left testis. Figure 2 shows the results of computed tomography (CT) imaging findings of the liver of the patient. CT revealed a mass of approximately 8.8 × 7.0 cm in the right lobe of the liver. The resected testis was approximately 4 × 3 × 2 cm with a local thickened capsule and no notable changes in the cut surface of the testis. No abnormity was detected in the descended testis. Chest X-ray was performed and shows no abnormity.

**Fig. 3:**
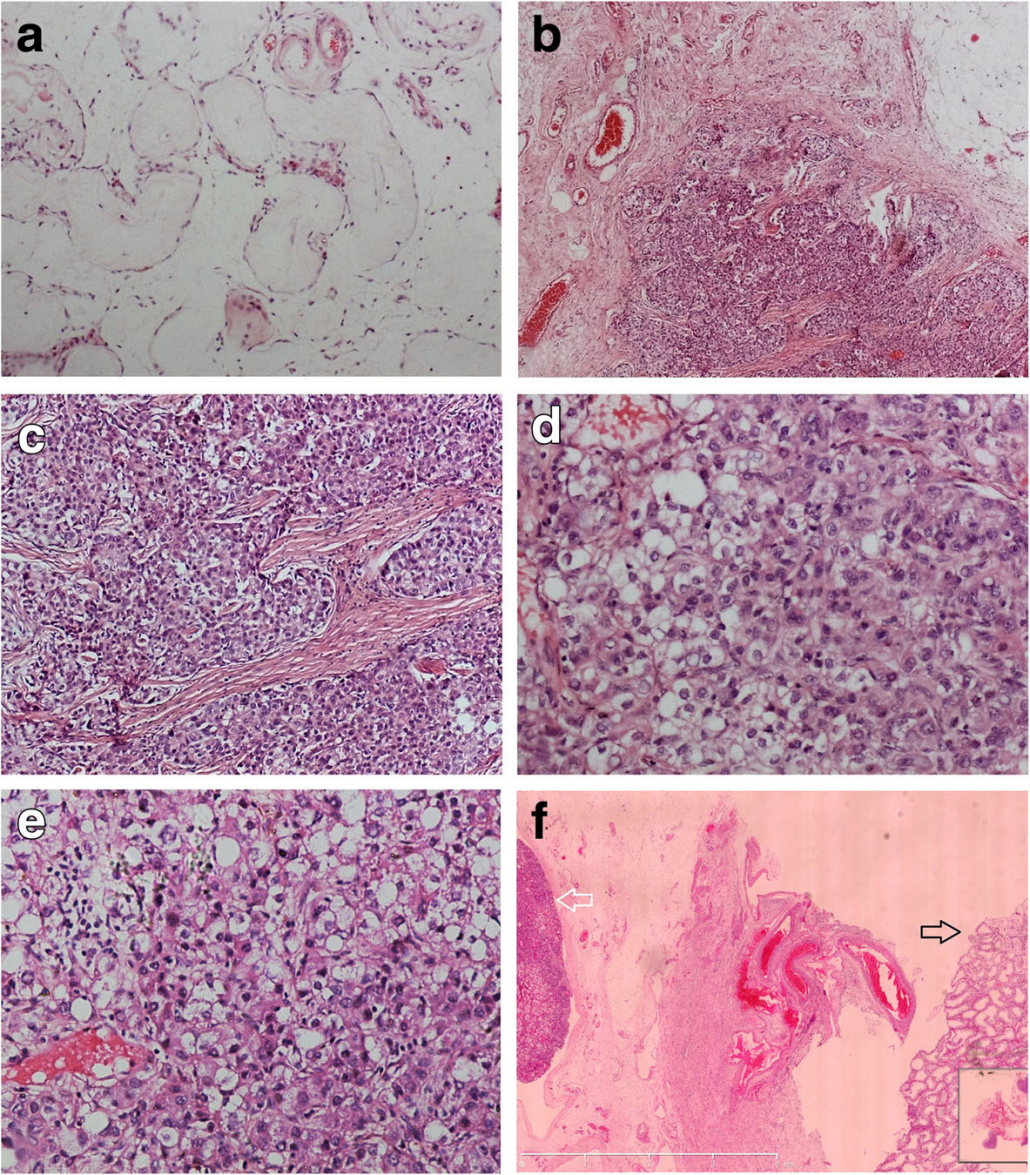
Morphologic features of the testis and the tumor. The tissues of the testis in the right inguinal region showed seminiferous tubular atrophy and transparent degeneration and thickening of the basement membrane (**a** × 200). No spermatogenic cell was detected in the seminiferous tubules. The cancer cells were located in the fat and fibrous tissues of the capsule of the testis and were arranged in irregular nests (**b** × 100; **c** × 200). The cancer cells were large and polygonal with abundant red or transparent cytoplasm; nuclear atypia was notable (**d**, **e** × 400). The location of the cancer cells and the adjacent testicular tissue was shown in **f** (× 4000)

The histopathological findings of the undescended testis and the tumor in the capsule of the testis can be observed in Fig. 2. The tissues of the testis in the right inguinal region showed seminiferous tubular atrophy and transparent degeneration and thickening of the basement membrane (a). No spermatogenic cell was detected in the seminiferous tubules. These findings are in accordance with the known and characterized histological features of an undescended testis in adults. The cancerous cells were located in the fat and fibrous tissues of the capsule of the testis and were arranged in irregular nests (b, c). They were large and polygonal with abundant red or transparent cytoplasm (d, e), and nuclear atypia was notable. We found an abundance of small blood vessels in the carcinoma tissues. There were only a few cancer cell nests in the capsule around the testis and no cancerous tissues were found inside the testis. Figure 3f shows the location of the cancer cells and the adjacent testicular tissue.

**Fig. 4:**
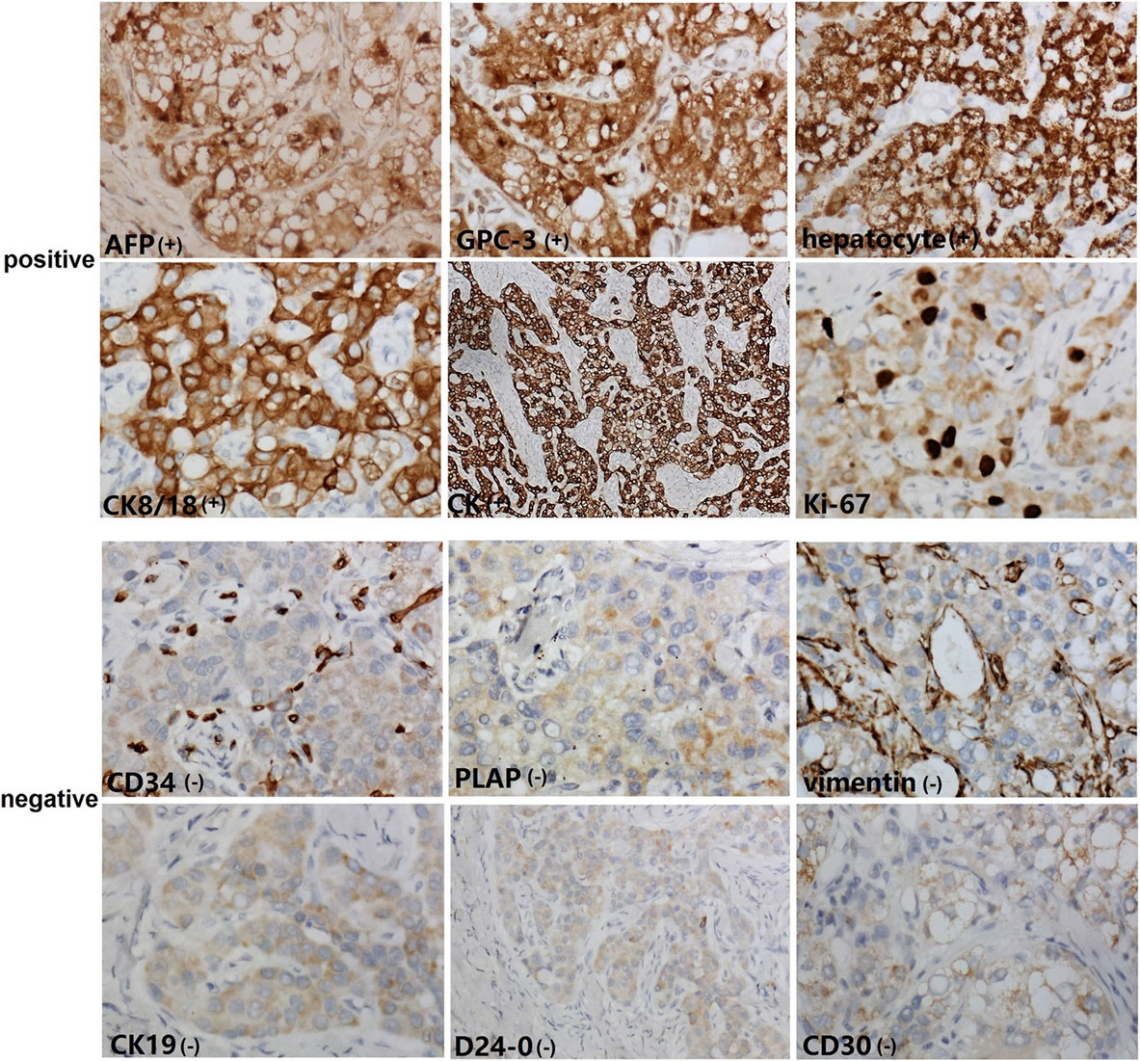
Immunohistochemical staining of the tumor. The tumor cells were diffusely positive for AFP, CK, CK8/18, GPC-3, and hepatocyte, but negative for CD30, CD34, CK19, D2–40, PLAP, and vimentin. The Ki-67 index was approximately 10% (D2–40, × 200; the others: × 400)

The tumor cells were diffusely positive for AFP, CK, CK8/18, GPC-3, and hepatocyte, but negative for CD30, CD34, CK19, D2–40, PLAP, and vimentin. The Ki-67 index was approximately 10% (Fig. 4). CD34 immunostaining indicated abundant small blood vessels in the cancer tissues. The immunohistochemistry of the samples was performed according to the instruction the producer. Negative control was obtained by omission of the primary antibodies.

The patient was treated with chemotherapy (5-FU + DDP). No more metastasis was detected 6 months after the therapy. AFP level reduced markedly after the chemotherapy (< 500 ng/mL). The tumor shrunk to 6.8 × 6.1 cm in size. The tumor metastasizing to the inguinal area was totally resected and had no relapse.

This study was approved by the institutional Ethics Committees of China Medical University and conducted in accordance with the ethical guidelines of the Declaration of Helsinki.

## Discussion

HCC is a common malignancy worldwide and the incidence has been rising (from 1.6/100000 to 4.6/100000) [8]. Here, we report a case of metastatic HCC presenting in an extremely rare site in a patient who had a unique clinical history. The patient was found to have cryptorchidism and the undescended testis in his right inguinal region was not resected prior to reporting to our hospital. Cryptorchidism is found in roughly 1–3% of full-term newborns [9] and approximately 1% of 1-year-olds [10]. The undescended testicles stay in the inguinal region or in the abdominal cavity. The risk of germ cell tumors increases in people with cryptorchidism compared to those without the disease [11]. Seminoma is the most common type of germ cell tumor in the testes [12]. The most common sites of hematogeneous spread of seminoma include the liver, lung, and bones [13]. In this case, the tumor in the liver was originally considered a metastatic tumor from the undescended testis. Guo reported a case of seminoma which metastasized to the liver and neck [12]. In this case, the patient underwent an orchidectomy of the testis in his right inguinal region. The tumor was located in the capsule of the testis and the histological and immunostaining findings proved the mass to be a metastatic HCC but not seminoma. The tumor cells were positive for AFP, a useful marker for early tumor detection of hepatocellular carcinoma. However, this marker is usually negative in seminoma [13]. GPC-3 and hepatocyte are both specific markers for hepatocellular carcinoma, which were also positive in the tumor cells in the current case. On the contrary, PLAP is usually positive in seminoma but not hepatocellular carcinoma and was negative in the current case. These findings all support the diagnosis of hepatocellular carcinoma.

HCC mostly invades via the portal vein system and spreads in the liver [1]. Distant metastases are mostly found in the lungs [1]. Rare sites of distant metastases include the jaw, skull, skin, and mandible to name a few [2–5]. HCCs that metastasize to the testis are also rather rare. Young reported a case of HCC which metastasized to the testis in a 57-year-old man [6]. Yamauchi reported a case of double cancer (hepatocellular carcinoma and mixed germ cell tumor of the testis) in a 43-year-old male [14]. In this case, the HCC metastasized to the inguinal area adjacent to the undescended testis and it was located in the capsule of the testis but not inside the testis. Cancer cells may be involved in the inguinal area after invading the peritoneum first. HCC metastasis to the inguinal area is also rare and only one case of an HCC that had metastasized to the spermatic cord of the inguinal region has been reported [15]. In this particular case, the HCC also metastasized to the inguinal region, where the undescended testis co-localized. There was not a clear mass in the testis, and only an abnormal signal in the capsule was detected via ultrasound. A few cancer cell nests were detected under the microscope in the capsule adjacent to the undescended testis. The histological findings of the testis were consistent with the features of an undescended testis. However, no seminoma was detected in the testis. A high serum AFP level is useful for predicting of HCC [8]. In this case, the AFP level of the patient was markedly elevated (> 1210 ng/mL). Korenbaum reported an unusual case of hepatocellular carcinoma which was a second cancer after radiotherapy for testicular seminoma [16]. In this case, the patient had not received any chemotherapy before the tumor in the liver was detected. According to the results of these findings, the tumor in the capsule of the undescended testis was determined to be a metastasized HCC, rather than seminoma, which can metastasize to the liver. Retroperitoneal lymph nodes, lung, and liver are the most common site of the metastasis of testicular seminoma [17–19]. There were also some complicated and unusual conditions related to testicular seminoma reported. Akin reported an extremely rare case of testicular seminoma accompanied by Wilson disease. Wilson disease caused liver cirrhosis which was suspected as metastasis of seminoma in the patient [20]. Tumor-to-tumor metastases have also been documented. Ro reported a rare case of lung carcinoma which metastasized to testicular seminoma [21]. Thus, the patients need a thorough examination to exclude the possibility of other tumors when metastasis of testicular seminoma is suspected. The cryptorchidism in this case is unilateral. Salman’s study indicated that the undescended testis impeded the germinal epithelium maturity in the contralateral descended testicle in mice [22]. Another paper indicated that orchiectomy for unilateral cryptorchidism was helpful for the tissue development of the contralateral testis in mice [23]. But, as no mass was detected by ultrasonography in the descended testis in the current case, pathological examination was not performed on it. However, the ultrasonography showed a relatively smaller testis in the left scrotum, which indicates possible hypoplasia of the testis.

## Conclusion

HCCs that metastasize to the inguinal region or testis are both rather rare. Here, we report an exceptionally rare case in which an HCC metastasized to the capsule of the undescended testis located in the inguinal region. This exceedingly rare case illustrates the fact that during the diagnosis of malignancies, metastatic tumors should always be considered in the differential diagnosis even if they present in rare metastatic sites or concurrently with other disease(s).
